# CRISISS: A Novel, Transcriptionally and Post-Translationally Inducible CRISPR/Cas9-Based Cellular Suicide Switch

**DOI:** 10.3390/ijms24129799

**Published:** 2023-06-06

**Authors:** Maximilian Amberger, Esther Grueso, Zoltán Ivics

**Affiliations:** Research Center, Division of Hematology, Gene and Cell Therapy, Paul-Ehrlich-Institute, 63225 Langen, Germany; maximilian.amberger01@web.de (M.A.); esther.grueso@ufv.es (E.G.)

**Keywords:** suicide switch, suicide gene, CRISPR/Cas9, *Alu* retrotransposon, *Sleeping Beauty* transposon, gene therapy

## Abstract

With the ever-increasing developing rate of gene and cellular therapy applications and growing accessibility due to products receiving regulatory approval, the need for effective and reliable safety mechanisms to prevent or eliminate potentially fatal side effects is of the utmost importance. In this study, we present the CRISPR-induced suicide switch (CRISISS) as a tool to eliminate genetically modified cells in an inducible and highly efficient manner by targeting Cas9 to highly repetitive *Alu* retrotransposons in the human genome, causing irreparable genomic fragmentation by the Cas9 nuclease and resulting cell death. The suicide switch components, including expression cassettes for a transcriptionally and post-translationally inducible Cas9 and an *Alu-*specific single-guide RNA, were integrated into the genome of target cells via *Sleeping-Beauty-*mediated transposition. The resulting transgenic cells did not show signs of any impact on overall fitness when uninduced, as unintended background expression, background DNA damage response and background cell killing were not observed. When induced, however, a strong expression of Cas9, a strong DNA damage response and a rapid halt of cell proliferation coupled with near complete cell death within four days post-induction were seen. With this proof-of-concept study, we present a novel and promising approach for a robust suicide switch with potential utility for gene and cell therapy in the future.

## 1. Introduction

Genetic engineering technologies developed and polished in recent times have succeeded in gaining a foothold and opening a new chapter in modern medicine. Genetic modifications, especially those technologies that introduce novel genetic information to equip cells with new functions, can convert cells into “living drugs”, enabling treatment and the correction of previously untreatable conditions. Most prominently, adoptive T cell immunotherapy has seen a recent breakthrough with several products approved for therapeutic use by the Food and Drug Administration and the European Medicines Agency, with multiple additional candidates going through clinical trials in order to gain approval.

Widely regarded as a powerful tool for cancer treatment, these cellular products share within their manufacturing process the genomic integration of a therapeutic gene into their genome. As this integration step typically occurs in a non-specific and non-targeted manner, it is accompanied by the possibility of unintentionally disrupting, upregulating or downregulating gene expression patterns, potentially leading to detrimental effects including the risk of causing malignant transformation, as demonstrated recently for the first time in a clinical trial employing chimeric antigen receptor (CAR)-T cells [1]. Additionally, severe side effects including neurotoxicity and cytokine release syndrome (CRS) can pose a severe threat to patients undergoing therapy [2]. Furthermore, not only additional adoptive T cell therapies targeting cancer are advancing rapidly from preclinical to clinical settings, but also treatments regarding the correction of monogenic disorders, often requiring the integration of foreign DNA into the target cell’s genome. Thus, the genotoxic risks elicited via vector integration described above are likely to manifest, as this technology is incorporated in further treatments, and previously rarely observed occurrences could become more frequent. Another area of cell therapy with tremendous potential in regenerative medicine relies on differentiating therapeutically applicable cell types from induced pluripotent stem cells (iPSCs) in vitro, followed by the transplantation of the cells in the hope of a regenerative, therapeutic impact. However, the possibility for oncogenic transformation remains a potential risk for the safe use of iPSCs in regenerative medicine. The risk of oncogenesis stems from genetic and epigenetic instability during the reprogramming and expansion of iPSCs in vitro [3], the presence of partially or abnormally reprogrammed cells in iPSC populations or teratoma formation from a small number of pluripotent cells remaining in transplanted iPSC-derived populations. For both genetically engineered gene therapy applications, including CAR-T cell products [4], as well as iPSC-based cell therapy approaches, the implementation of safeguarding mechanisms is quite likely a critical component for the advancement of these cells into clinical applications [5,6].

Suicide switches are a feature that can be used to irreversibly remove genetically modified cells from a patient by installing a final exit option into a therapeutic cell product in case the potential adverse effects outweigh the product’s benefit. Indeed, multiple approaches include them in their transgenic vector design so that they are integrated alongside the therapeutic gene, effectively equipping every modified cell with it. Importantly, the switch remains with the cell for its lifetime, thereby providing a lifelong option for their elimination, if necessary. Prominent suicide switch technologies include inducible apoptotic genes such as iCasp9 [7], metabolic enzymes converting nontoxic prodrugs into toxic compounds such as the herpes simplex thymidine kinase (HSV-TK) [8] and cytosine deaminase (CD) [9] or epitope-based approaches by expressing a peptide on the cell’s surface that can be targeted by administering a peptide-specific therapeutic monoclonal antibody [10]. However, all of these approaches come with distinct disadvantages, which include incomplete cell ablation [11], toxic effects of the prodrug [12], immunogenicity [13], cell cycle dependence [14] and worsening of and/or causing CRS [15].

Here, we present a novel CRISPR/Cas9-based inducible suicide switch that can be stably integrated into the host cell’s genome and which exhibits rapid and efficient cell killing within 4 days without affecting overall cell fitness when uninduced. The system combines the post-translational control of an allosterically inducible Cas9 enzyme [16] with the transcriptional control of a Tet-on system [17]. Cell killing is achieved by targeting this inducible CRISPR/Cas9 system to *Alu* retrotransposons, which make up a significant portion of the human genome [18] via *Alu*-specific single-guide RNAs (sgRNAs). Using CRISPR/Cas9 in this unconventional way, maximizing target site number and disregarding off-target effects, the host cell’s genome is irreversibly fragmented and cell death is inevitable.

## 2. Results

### 2.1. Multi-Component CRISISS System Enables Tight, Doxycycline-Dependent Transcriptional Regulation of arC9 Expression

**Figure 1:**
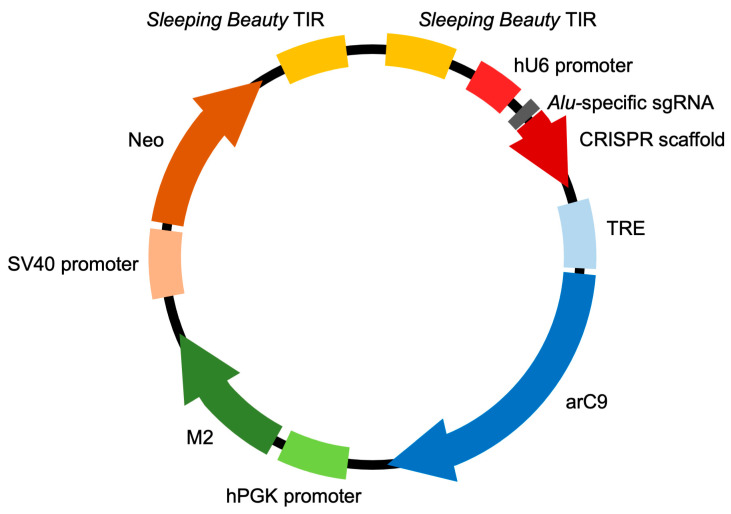
The CRISISS inducible cell killing system. Schematic overview of the composition of the pTOV_T11_Neo_arC9_Alu plasmid, carrying all CRISISS components including hU6 promoter-driven single-guide (sg) RNA-targeting human *Alu* retrotransposons, a tetracycline response element (TRE)-driven, allosterically regulated Cas9 (arC9) gene, a human PGK (hPGK) promoter-driven reverse transcriptional transactivator (M2) and an SV40 promoter-driven neomycin resistance gene (neo), all between the flanking terminal inverted repeat (TIR) sequences of the SB transposon. The arc9, M2 and neo coding sequences are followed by polyadenylation sequences, which were omitted from this figure for the sake of simplicity.

**Figure 2:**
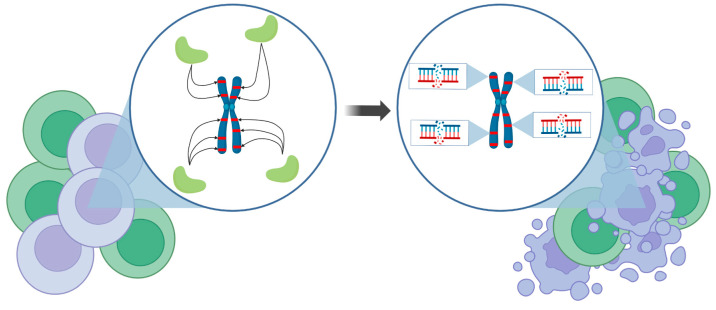
Conceptual depiction of CRISISS’ mode of action. Upon induction, genetically modified cells (violet cells), carrying the CRISISS suicide cassette, express the Cas9 protein (light green shapes) armed with *Alu*-specific sgRNAs, which enable specific cleavage at the repetitive *Alu* retrotransposons in the human genome (red stripes). Only a handful of *Alu* elements are depicted here for the sake of clarity and simplicity. This process leads to an irreparable amount of DSBs and subsequently to the specific killing of genetically modified cells without disturbing the unmodified cells (green cells). Figure made using BioRender.com.

We generated a highly complex, multi-genic *Sleeping Beauty* (SB) transposon vector (Figure 1) containing the following functional elements: (1) a U6 promoter-driven sgRNA against a conserved region of the human *Alu* retrotransposon, previously validated for efficiently guiding Cas9 to the targeted sites [19], (2) an engineered, allosterically switchable Cas9 (arC9) containing the Human Estrogen Receptor α Ligand-Binding Domain (ER-LBD) [16] under the transcriptional control of the tetracycline response element (TRE) [17], (3) a PGK promoter-driven reverse (Tet-On) M2 transactivator [20] and (4) an SV40 promoter-driven neomycin resistance gene. The four expression cassettes are flanked by the terminal inverted repeats (TIRs) of the SB transposon, so that transposition out of this plasmid in the presence of the SB transposase results in the genomic integration of all four genes in a single genetic engineering step. In cells transgenic for transposon insertions, arC9 expression can be transcriptionally triggered by adding doxycycline (DOX) to the culture medium, which allows for the binding of the M2 transactivator to the TRE, whereas the allosteric switch that allows for post-translational activation of arC9 is regulatable by the drug 4-hydroxytamoxifen (4-HT). Once an active Cas9 nuclease is present in the nucleus of the cell, it is expected to be guided by the specific sgRNA to the *Alu* retrotransposon target sequences endogenous in the human genome, and double-strand breaks (DSBs) are introduced in those sites. Because there are thousands of *Alu* elements scattered around the human genome, cleaving the DNA at these sites is expected to result in effective and irreparable fragmentation of the genome, resulting in cell death (Figure 2). We named this multi-component suicide switch machinery CRISPR-induced suicide switch (CRISISS).

**Figure 3:**
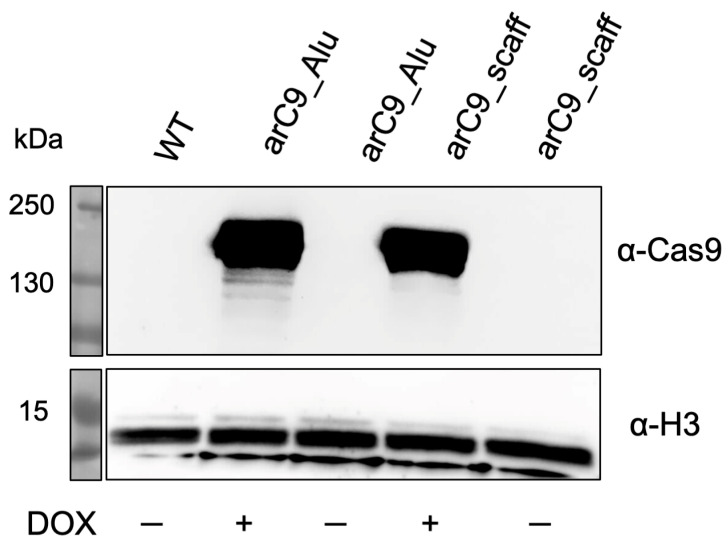
Doxycycline-inducible expression of arC9 in transgenic HeLa cells harboring the CRISISS cassette. Western blot analysis of cell extracts generated from established polyclonal HeLa cell lines harboring CRISISS with (arC9_Alu) and without (arC9_scaff) *Alu*-specific sgRNAs. A cell extract stemming from wild-type (WT) HeLa cells was used as a negative control. As a loading control, H3 was detected. For arC9 detection, the membrane was exposed for a total of 5 min; for loading control detection, the membrane was exposed for a total of 9 s.

We co-transfected the CRISISS vector together with an SB100X transposase expression plasmid into human HeLa cells, and put the cells under G418 selection in order to allow the formation of cell colonies harboring genomic integration of the CRISISS cassette. G418-resistant cell colonies were pooled to generate polyclonal transgenic cell lines with (arC9_Alu) and without (arC9_scaff) *Alu*-specific sgRNAs. In order to determine DOX-dependent arC9 expression and assess the leakage levels in the absence of DOX, cell extracts generated 48 h post-induction with DOX from the established polyclonal cell lines were subjected to Western blot analysis, alongside samples extracted under uninduced conditions and wild-type HeLa cell extract as a control. Hybridization with a Cas9-specific antibody showed that the cell lines arC9_Alu as well as arC9_scaff exhibited a strong, DOX-dependent expression of arC9, while no apparent transcriptional leakage was detectable when the transcriptional inducer was absent (Figure 3). Given these results, monoclonal cell lines were generated for downstream experiments.

### 2.2. CRISISS-Mediated DNA Damage Is Dependent on sgRNA Presence and Switch Induction

In response to DNA damage, members of the Phosphatidylinositol 3-kinase-related kinase (PIKK) family phosphorylate KRAB domain-associated protein 1 (KAP1) at the serine residue in position 824 (S824). pKAP1 co-localizes with several DNA repair factors, including γH2AX, implicating a role for KAP1 in DNA repair processes [21]. Furthermore, the phosphorylation of KAP1 at S824 is responsible for ATM-mediated chromatin relaxation, a crucial step for DNA DSB repair [21].

**Figure 4:**
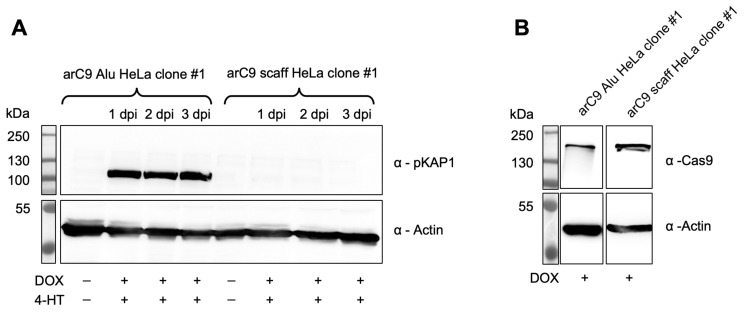
CRISISS triggers DNA damage response in an inducible and sgRNA-dependent manner. (**A**) Western blot analysis on protein extracts of monoclonal cell lines harboring CRISISS with (arC9 Alu HeLa clone #1) and without (arC9 scaff HeLa clone #1) *Alu*-specific sgRNAs over an induction period of 72 h with 4-HT and DOX to detect pKAP1 in response to CRISISS-induced DNA damage. (**B**) Western blot analysis on protein extracts of monoclonal cell lines harboring CRISISS with (arC9 Alu HeLa clone #1) and without (arC9 scaff HeLa clone #1) *Alu*-specific sgRNAs after 48 h induction to confirm DOX-dependent arC9 expression.

Since CRISISS was designed to induce fatal levels of DNA damage in the form of DSBs to cells carrying the switch while being inactive when uninduced, Western blot analysis was performed to detect the presence of pKAP1 protein upon transcriptional and post-translational induction with DOX and 4-HT. pKAP1 was detected exclusively in the monoclonal transgenic cell line arC9 Alu HeLa clone #1 at the earliest timepoint of 1 day post-induction (dpi) and was continuously detected until the end of the assay at 3 dpi (Figure 4A). The levels of pKAP1 did not visibly decrease or increase after being first detected at the 1 dpi timepoint, suggesting that DNA damage reached critical levels between 0 and 24 h post-induction. This not only confirms CRISISS-induced DNA damage, but it also strongly suggests early cell cycle arrest and/or induction of apoptosis. When induction with DOX and 4-HT was omitted, no detectable levels of pKAP1 were observed. Similarly, no traces of pKAP1 were detected in the control cell line arC9 scaff HeLa clone #1, which lacks *Alu*-specific sgRNAs, irrespective of timepoint and inducer presence, despite exhibiting similar arC9 expression levels when induced with DOX (Figure 4B). In conclusion, CRISISS induces DNA damage strictly dependent on the presence of *Alu-*specific sgRNAs.

### 2.3. Near-Complete Cell Killing upon CRISISS Activation

After confirming the functional transcriptional control of arC9 expression and a CRISISS-induced DNA damage response dependent on the presence of *Alu*-specific sgRNAs when fully induced with 4-HT and DOX, growth assays were performed in order to assess CRISISS-induced cell killing upon activation.

**Figure 5:**
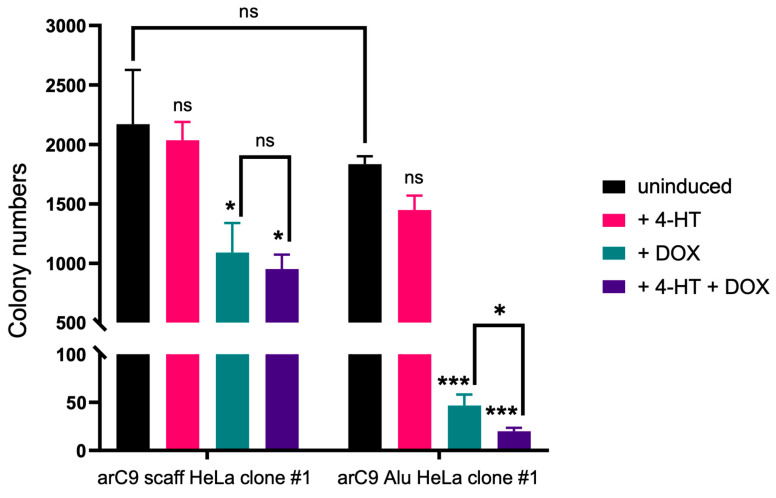
CRISISS triggers cell killing in an inducible and sgRNA-dependent manner. Shown are the colony numbers corresponding to the monoclonal cell lines harboring CRISISS with (arC9 Alu HeLa clone #1) and without (arC9 scaff HeLa clone #1) *Alu*-specific sgRNAs on a 10 cm cell culture dish within 4 dpi of single induction with 4-HT (pink) or DOX (petrol), double induction with 4-HT and DOX (violet) and uninduced (black). Significances were calculated with respect to the corresponding negative control (uninduced sample, black). Error bars: SD, * = *p* < 0.05, *** = *p* < 0.001, ns = not significant.

No significant change in cell numbers and thus CRISISS leakage was observed when comparing the growth rates and survival of uninduced samples of both cell lines (Figure 5). Additionally, cells lacking *Alu*-specific sgRNAs did not exhibit a significant growth disadvantage when induced with 4-HT only. In contrast, cells harboring CRISISS with *Alu*-specific sgRNAs exhibited a significant level of reduction in survival rate when compared to the uninduced sample (Figure 5). Additionally, despite not harboring sgRNAs, cells undergoing single induction with DOX and double induction with 4-HT and DOX showed a significant reduction in cell survival rate when compared to the uninduced sample. Because there is no significant impact of DOX on HeLa cell viability, and because cells lacking *Alu*-specific sgRNAs do not mount a DNA damage response upon the induction of arC9 expression (shown above), we hypothesize that the mere overexpression of the arC9 protein has a negative impact on cellular fitness. Most importantly, however, the most striking differences were observed within samples harboring *Alu*-specific sgRNAs when undergoing single induction with DOX and double induction with DOX and 4-HT. In both samples, colony numbers decreased by ~97.5% and ~99%, respectively, demonstrating efficient cell killing by the CRISISS system (Figure 5). Interestingly, DOX alone was sufficient to induce cell killing in this setup, and the addition of 4-HT had only a slight, yet statistically significant, impact.

### 2.4. CRISISS Killing Kinetics Exhibit a Rapid Arrest of Cell Proliferation Followed by Near-Complete Cell Ablation

Lastly, the killing kinetics upon CRISISS activation was investigated. For this purpose, the cell growth of two monoclonal cell lines harboring CRISISS with and without *Alu*-specific sgRNAs was monitored alongside wild-type HeLa cells used as a control over a period of 7 days, during which the inducers 4-HT and DOX were present in the growth medium.

**Figure 6:**
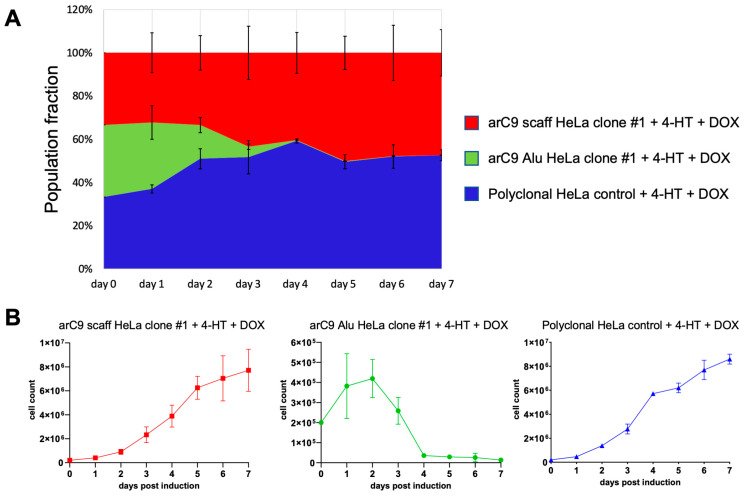
Kinetics of CRISISS-induced cell killing. The killing kinetics of the CRISISS system was monitored over an induction period of 7 days. (**A**) Representation of the relative fraction of living cells in each population as compared to the starting cell numbers, which was set to 100%, after induction with 4-HT and DOX. Each cell line started with an equal starting condition of 2 × 10^5^ cells on day 0. (**B**) Numbers of total living cells of each replicate of each individual cell line, determined daily over the entirety of the induction period. Significances were calculated with respect to the data of the arC9 scaff HeLa clone #1 cell line (red line).

As seen in Figure 6, the control cell lines arC9 scaff HeLa clone #1 and wild-type HeLa cells both exhibited exponential growth until plateauing at about an average total cell number of 6 × 10^6^ cells per well, due to the limits imposed by the physical size of the 6-well plate. In stark contrast, the monoclonal cell line arC9 Alu HeLa clone #1 significantly slowed down cell divisions as early as on day 2 post-induction in comparison to both control cell lines (Figure 6A,B). Cell numbers started to decrease on day 3 post-induction, and on day 4 post-induction, only 0.37% surviving arC9 Alu HeLa clone #1 cells were left in the experiment. The cell numbers further declined until the end of the experiment at 7 dpi and did not recover or show any signs of cell division. In summary, the CRISISS system enables near-complete elimination of cells within 4 days of transcriptional and post-translational induction.

## 3. Discussion

Inducing cell death via the means of producing a high number of DSBs is a procedure that has a long history in medicine. Alongside chemotherapy and surgery, radiotherapy is a pillar of current cancer treatment. By focusing ionizing radiation onto cancerous tissue, critical numbers of DSBs are generated (reviewed in [22]). To prevent genomic instability, if the DNA damage exceeds the potential of the cell’s DNA repair mechanisms, the p53 pathway leads to an accumulation of pro-apoptotic proteins that trigger programmed cell death (reviewed in [23]). Independently from the work presented here, other groups have recently succeeded in targeting *Alu* elements with CRISPR/Cas9 with the purpose of inducing cell killing [24,25]. By supplementing Cas9 with *Alu-*specific sgRNAs, they were able to efficiently kill cancer cells in proof-of-concept studies, presenting the possibility of using this method to target difficult-to-treat cancer types. However, for the intended purposes of this work, their design does not represent an option for a suicide switch, since the key feature of inducibility is missing. Thus, to fulfil the requirements for a working suicide switch, a major challenge of this work was to engineer an *Alu-*targeted CRISPR/Cas9 system that can be stably introduced into a target cell, persists for an indefinite time without exhibiting unintended leakage and presents reliable inducibility leading to the complete clearance of cells harboring the switch.

In this proof-of-concept study, we show that our novel CRISISS system functions as a highly efficient suicide switch that can be stably introduced into the genomes of target cells via non-viral, SB-mediated transposition. To our knowledge, this is the first time that CRISPR/Cas9 technology has been used to function as an inducible suicide switch. By employing a combination of transcriptional and post-translational control, we ensure robust inducibility leading to a rapid halt in cell proliferation and a subsequent, near-complete killing of target cells, coupled with unmeasurable levels of leakage when uninduced. Importantly, the latter was confirmed via three independent experiments where cells harboring CRISISS did not exhibit measurable Cas9 expression (Figure 3), DNA damage response (Figure 4) or significant differences in cell proliferation (Figure 5 and Figure 6) in an uninduced state. Of note, the transcriptional control of arC9 in our approach does not serve the sole purpose of the drug-inducible supply of the Cas9 nuclease, but simultaneously eliminates a potential immunogenicity issue that would be expected when arC9 is expressed in vivo under the regulation of a constitutive promoter [26]. The experiments presented in Figure 6 resulted in marginal numbers of surviving cells at the end of the observation period (7 days post-induction). Importantly, however, these remaining live cells displayed senescent phenotypes without exceptions. For a suicide switch system, this proves highly valuable as, while close-to-complete cell killing was achieved, the remaining cells lose their proliferative properties and cannot escape. In an in vivo scenario it would thus be likely highly beneficial for two main reasons: (i) considering the ultimate threat of malignant transformation of a gene therapeutic cell product, CRISISS showed that within the context of a cancer cell line (HeLa) as an in vitro model, besides near-complete cell clearance, a potent tumor-suppressive state (reviewed in [27,28]) was achieved in the remaining cells, completely stopping proliferative properties, and (ii) the general coupling to a senescence-associated secretory phenotype [29] would suggest swift clearance by the immune system in vivo (reviewed in [30,31]) by recruiting macrophages, natural killer cells, neutrophils and T lymphocytes via pro-inflammatory protein secretion [32]. The CRISISS system thus effectively addresses the shortcomings of established kill switch systems. First, exceptional levels of cell killing were achieved, coupled with no detection of surviving proliferative cells, strictly dependent on both essential components of the system: *Alu-*specific sgRNAs and the double induction of transcription and post-translational protein activation. Second, CRISISS carries the potential of being triggered in vivo by the small molecule inducers DOX and 4-HT which exhibit excellent biodistribution, tissue penetration and tolerability [33,34].

Future developments based on the present study’s reports might employ CRISISS alongside a therapeutic gene in primary cells to assess the performance of the system in a clinically relevant context. According to calculations, the human genome harbors a theoretical number of 284,559 target sites for the presented *Alu*-specific sgRNAs (Cas-OFFinder tool, [35]). Judging by CRISISS’ performance when tested within the context of the robust nature of HeLa cells used in this study, more sensitive cells such as primary cells are expected to react at least in a similar way to CRISISS’ DNA-damaging potential. Concerning the relatively large size of the transgene cargo of CRISISS that has to be introduced into a cell and then undergo genomic integration, the system was purposely designed to rely on the SB transposon that allows greater cargo capacity and simpler handling when compared to viral methods. Importantly, SB transposition allows for theoretical transposition of sequences reaching 100 kb in length [36], and transposition efficiency can be greatly aided by the employment of minicircle technology [37], which can be employed to aid CRISISS delivery in therapeutically relevant, primary human cells. Lastly, both inducers needed for CRISISS activation are administrable in vivo. Future studies might validate CRISISS in an in vivo setting by transplanting cells harboring the system and clearing the animal of modified cells via the feeding/injection of inducers. With the data presented here, we strongly believe that CRISISS has the potential to circumvent current suicide switch shortcomings, especially incomplete cell ablation, cell cycle and cell type dependence, and encourage future developments aiming at establishing CRISISS as a viable suicide switch option.

## 4. Materials and Methods

### 4.1. Cloning of the SB Transposon Vector Containing CRISISS

An hU6 promoter-driven CRISPR scaffold sequence [38] was PCR-amplified (forward primer: 5′-TTTGTCGACATGGATTACAAAGACGATGACG-3′, reverse primer: 5′-TTTGCGGCCGCTCAGGATTCGGATCCGTCG-3′), introducing restriction sites *Nhe*I and *Age*I at the 5′- and 3′-end, respectively, and cloned into the pTOV_T11_Neo plasmid [20] between the restriction sites *Nhe*I and *Age*I. The arC9 gene [16] was PCR-amplified (forward primer: 5′-TTTGCTAGCTTTCCCATGATTCCTTCATATTTGC-3′, reverse primer: 5′-TTTACCGGTAAAAAAAGCACCGACTCGG-3′), introducing a stop codon and restriction sites *Not*I and *Sal*I at the 5′- and 3′-end, respectively, and cloned between restriction sites *Not*I and *Sal*I. Finally, the *Alu*-specific sgRNA sequence (5′-TCCCAAAGTGCTGGGATTAC-3′) [19] was introduced upstream of the CRISPR scaffold sequence between *Bbs*I restriction sites, completing the pTOV_T11_Neo_arC9_Alu plasmid. A similar version, lacking *Alu*-specific sgRNAs and serving as a control, was named pTOV_T11_Neo_arC9_scaff.

### 4.2. Generation of Polyclonal and Monoclonal HeLa Cell Lines Harboring CRISISS and Induction of Transgene Expression

HeLa cells were cultured in DMEM (Life Technologies, Carlsbad, CA, USA) containing 10% FBS (PAN-Biotech, Aidenbach, Germany) and 1% penicillin-streptomycin (in-house) at 37 °C and 5% CO_2_. CRISISS was integrated into the genome of HeLa cells via SB-mediated transposition. For this purpose, 2.5 × 10^5^ HeLa cells seeded on 6-well plates one day prior to transfection were co-transfected with 500 ng of the CRISISS-harboring plasmid pTOV_T11_Neo_arC9_Alu and 300 ng of the SB100X expression plasmid pCMV(CAT)T7-SB100X [39] using the transfection reagent LT1 (Mirus Bio, Madison, WI, USA) according to the manufacturer’s recommendations. Selection with 1 mg/mL G418 (InvivoGen, San Diego, CA, USA) was started 24 h post-transfection and continued for a period of two weeks, performing regular medium changes and passages when necessary. Following the G418 selection process to produce a polyclonal, G418-resistant HeLa cell pool harboring CRISISS termed arC9_Alu, a single-cell suspension was seeded at a density of 5 × 10^3^ cells/mL on a 10 cm cell culture dish. Cells were monitored daily under a light microscope until well-defined colonies, stemming from a single cell, were discernible. Individual colonies were picked using a micropipette, transferred to a 24-well plate and expanded. After expansion, the entire process of low-density seeding and colony picking was repeated once again to produce a G418-resistant, monoclonal HeLa cell line termed arC9 Alu HeLa clone #1. The process was repeated using the version of the CRISISS-harboring plasmid lacking *Alu-*specific sgRNAs to produce a polyclonal control cell line termed arC9_scaff and a monoclonal control cell line termed arC9 scaff HeLa clone #1. Doxycycline (DOX) (Fisher Scientific, Hampton, VA, USA) and 4-Hydroxytamoxifen (4-HT) (Sigma-Aldrich, St. Louis, MO, USA) were diluted in PBS and ethanol, respectively, and added directly to the cell culture medium when needed for CRISISS induction at a concentration of 1 µg/mL DOX and 200 nM 4-HT.

### 4.3. Detection of DOX-Dependent Cas9 Expression

Western blot (WB) analyses were conducted to confirm DOX-dependent arC9 expression. For this purpose, 2.5 × 10^6^ cells were seeded into wells of a 6-well plate and immediately induced by adding 1 µg/mL DOX. After 48 h, cells were washed with PBS, harvested and counted using an automated cell counter. The well containing the least amount of cells was taken as a reference, and the same amount of cells of each well was lysed in 250 µL RIPA buffer [1 mM EDTA, 50 mM HEPES (pH 7.8), 150 mM NaCl, 1% (*v*/*v*) NP-40, 0.25% (*v*/*v*) Na-deoxycholate, 99% ddH_2_O, 1X Protease-Inhibitor-Cocktail (Roche)] in a rotating incubator at 4 °C for 30 min. Cell debris were removed by sedimenting the samples at 14,000× *g* at 4 °C for 10 min and transferring 200 µL of the supernatant to fresh microcentrifuge tubes. Briefly, 50 µL of this supernatant corresponding to the extract of 2 × 10^5^ cells was mixed with 25 µL of 3X SDS sample-loading buffer [188 mM Tris-HCl (pH 6.8), 3% (*w*/*v*) SDS, 30% (*v*/*v*) glycerol, 0.01% (*w*/*v*) bromophenol blue, 15% (*v*/*v*) β-mercaptoethanol, 55% (*v*/*v*) ddH_2_O], incubated at 95 °C for 5 min and subjected to SDS-PAGE in an 8% gel. After electrophoresis, the proteins were transferred onto a nitrocellulose membrane at 90 V for 120 min. The membrane was blocked overnight at 4 °C in a rocking incubator with blocking buffer (5% milk in TBS-T). The membrane was cut and the halves were incubated at room temperature for 90 min with the primary antibody (top membrane half with α-Cas9 (Thermo Fisher, Waltham, MA, USA, RRID: AB_2610639) at a dilution of 1:5000 in blocking buffer, bottom half with α-H3 (Abcam, Cambridge, UK, RRID: AB_238971) at a dilution of 1:5000 in blocking buffer). After primary antibody incubation, the membrane halves were washed with TBS-T and incubated at room temperature for 60 min with the secondary antibody (top membrane half with α-mouse-HRP (Thermo Fisher, RRID: AB_228313) and bottom half with α-rabbit-HRP (Thermo Fisher, RRID: AB_228378) at a dilution of 1:5000 and 1:1500, respectively, in blocking buffer). After incubation with the secondary antibody, membranes were washed with TBS-T, developed using a chemiluminescence detection kit (Thermo Fisher) according to the manufacturer’s instructions and imaged using a CCD imager.

### 4.4. Detection of Phosphorylated KAP1 after CRISISS Induction

To confirm CRISISS-induced DNA damage by measuring the cellular DNA damage response, an assay was conducted to detect phosphorylated KAP1 protein after CRISISS induction. For this purpose, cells of the established monoclonal HeLa cell line harboring CRISISS with (arC9 Alu HeLa clone #1) and without (arC9 scaff HeLa clone #1) *Alu*-specific sgRNAs were induced with 200 nM 4-HT and 1 µg/mL DOX. Cells were harvested at timepoints 1 dpi, 2 dpi and 3 dpi alongside uninduced cells of both cell lines as controls and lysed in RIPA buffer for 30 min at 4 °C in a rotating shaker. Subsequently, a WB analysis was carried out in a similar way as described before with a few exceptions: (i) 5 µg of total protein was loaded onto the acrylamide gel per sample, (ii) a 10% polyacrylamide gel was used, and (iii) instead of milk, bovine serum albumin was used for the blocking buffer. The following antibodies were used: α-pKAP1 (phospho S824, Abcam, RRID: AB_70369) at a dilution of 1:1000 in blocking buffer, α-actin (Thermo Fisher, RRID: AB_2223496) at a dilution of 1:5000 in blocking buffer, α-rabbit-HRP (Thermo Fisher, RRID: AB_228378) at a dilution of 1:1500 in blocking buffer and anti-mouse-HRP (Thermo Fisher, RRID: AB_228313) at a dilution of 1:5000 in blocking buffer.

### 4.5. CRISISS Induction and Kill Assay

Briefly, 5 × 10^3^ cells of the cell lines arC9 Alu HeLa clone #1 and arC9 scaff HeLa clone #1 were seeded per induction condition in 10 cm cell culture dishes. For both cell lines, a total of four induction conditions were tested in triplicate: (i) uninduced, (ii) single induction with 200 nM 4-HT, (iii) single induction with 1 µg/mL DOX and (iv) double induction with 200 nM 4-HT and 1 µg/mL DOX. Inducers were added directly to the cell culture medium, which was regularly changed every 48 h for the entire induction period. At 5 dpi, cells growing on each cell culture dish were fixed with a 4% (*v*/*v*) PFA solution for 2 h at room temperature. After fixing, the PFA solution was replaced with a 0.2% (*w*/*v*) methylene blue solution, and plates were incubated for an additional 2 h. Afterward, the staining solution was removed, and the plates were rinsed with dH_2_O and dried overnight at room temperature. The plates were scanned and the software ImageJ (version 1.53f51) [40] was used to determine the colony numbers on each plate.

### 4.6. Determination of CRISISS Kill-Kinetics

Briefly, 2 × 10^5^ cells of the monoclonal cell lines arC9 Alu HeLa clone #1 and arC9 scaff HeLa clone #1, alongside polyclonal, wild-type HeLa cells (control), were seeded in the wells of 6-well plates, and inducers 4-HT and DOX were added to the cell culture medium at concentrations of 200 nM and 1 µg/mL, respectively. For a period of 5 dpi, for each cell line, the total numbers of living cells per well were determined after washing three wells per cell line with PBS to remove dead cells and cell debris, harvesting via trypsinization, resuspending them in cell culture medium to generate single-cell suspensions and counting them using an automated cell counter.

## Data Availability

The data presented in this study is contained within the article.
